# Unique charge-dependent constraint on collagen recognition by integrin α10β1

**DOI:** 10.1016/j.matbio.2016.08.010

**Published:** 2017-05

**Authors:** Samir W. Hamaia, Daisy Luff, Emma J. Hunter, Jean-Daniel Malcor, Dominique Bihan, Donald Gullberg, Richard W. Farndale

**Affiliations:** a Department of Biochemistry, University of Cambridge, Downing Site, Cambridge CB2 1QW, UK; b Department of Biomedicine, University of Bergen, Jonas Lies vei 91, N-5009 Bergen, Norway

**Keywords:** Integrin I domain, C2C12 cells, Cartilage, GROGER, Triple-helical peptides

## Abstract

The collagen-binding integrins recognise collagen through their inserted (I) domain, where co-ordination of a Mg^2 +^ ion in the metal ion-dependent site is reorganised by ligation by a collagen glutamate residue found in specific collagen hexapeptide motifs. Here we show that GROGER, found in the N-terminal domain of collagens I and III, is only weakly recognised by α10β1, an important collagen receptor on chondrocytes, contrasting with the other collagen-binding integrins. Alignment of I domain sequence and molecular modelling revealed a clash between a unique arginine residue (R215) in α10β1 and the positively-charged GROGER. Replacement of R215 with glutamine restored binding. Substituting arginine at the equivalent locus (Q214) in integrins α1 and α2 I domains impaired their binding to GROGER. Collagen II, abundant in cartilage, lacks GROGER. GRSGET is uniquely expressed in the C-terminus of collagen II, but this motif is similarly not recognised by α10β1. These data suggest an evolutionary imperative to maintain accessibility of the terminal domains of collagen II in tissues such as cartilage, perhaps during endochondral ossification, where α10β1 is the main collagen-binding integrin.

## Highlights

• Integrin α10β1 binding to collagen is mapped onto Collagen Toolkits.
• Charged residue in α10 I domain clashes with some binding sites that are unique to collagen II.
• Mutant constructs of other integrin I domains mimic this charge effect.
• Implications for evolution of collagens and cartilage with reference to bone formation

## Introduction

The collagen-binding integrin family is complete, with four established members, α1β1, α2β1, α10β1 and α11β1. These are highly-conserved, heterodimeric receptors with an important structural feature in common, the presence of an I (inserted) domain which contains the metal ion-dependent adhesion site (MIDAS). The MIDAS is a constellation of electronegative residues at the distal surface of this 200-residue domain that, in the resting, non-ligated state, can co-ordinate a Mg^2 +^ ion. Upon ligation, the octahedral co-ordination shell surrounding the metal ion is perturbed and reorganised by an incoming ligand, a negatively-charged glutamate residue that is crucial to the tight ligation of the receptor [1]. Several extracellular matrix (ECM) molecules are established as ligands for this group of integrins, including collagens, laminin, thrombospondin and others [2], [3]. However, it is as collagen receptors that the group is best known, with collagen I being a preferred ligand for α2β1, and collagen IV for α1β1, receptors that are considered the most abundant and widespread of the family [4]. Although the group as a whole is quite well conserved, α1 and α2 share greater homology with one another than with α10 and α11, and vice versa. However, the propensity for ligand binding runs counter to sequence homology, with α1β1 being more closely allied with α10β1, and α2β1 with α11β1 likewise [4], [5]. The family has been reviewed in depth recently [6], and needs little detailed introduction here.

Understanding of integrin activation advanced with the elucidation of a co-crystal structure comprising the α2 I domain and a synthetic triple-helical peptide (THP) containing the (now canonical) motif, GFOGER [7]. This hexapeptide motif is considered the highest affinity ligand present in collagens I and II, but also occurs in collagen IV and elsewhere. Many related motifs have now been identified using the Collagen Toolkits developed in this laboratory for the purpose [8]. These are libraries of overlapping peptides of 27 amino acids of collagen primary sequence (guest sequence), flanked by model [GPP]_5_ extensions (host sequence) that adopt triple-helical conformation, and so ensure that the quite diverse guest sequences are presented in their native conformation. The Toolkit approach revealed the presence in collagens II and III of several Gxx′GEx″ motifs with varying affinity for the collagen-binding integrins, depending also on the activation state of the integrin [9]. Thus, x is typically a long hydrophobic residue, such as the F residues from two chains of the GFOGER triple helix, which nestle against hydrophobic dimples in the α2 I domain surface. Unexpectedly, GROGER proved to be quite a good ligand, too, so that the aliphatic stem of the arginine sidechain appears capable of fulfilling the same role [8], [10]. x′ is usually hydroxyproline (O), the one exception to date being S, found in the weak ligands GLSGER in collagen III and GASGER in collagen I [11]. Although x″ is usually R, which forms a salt bridge with the I domain surface, integrin α1β1 is less discriminatory of the x″ position, since GLOGEN, occurring in collagen III, is a high-affinity ligand, and GVOGEA, from collagen II, is a moderate ligand that shows little affinity for α2β1 [12]. A low-resolution structure of a GLOGEN–α1 I domain has recently proven a similar mode of binding, that differs little in other respects from the structure of the GFOGER-α2 I domain complex [13].

Much less is known about the ligand propensity of α10β1, and it is with this topic in view that the present study was designed. In vivo, α10β1 appears to be expressed primarily on the chondrocyte surface but also in some junctional fibroblasts, in chondrogenic mesenchymal stem cells, and in cells lining endosteum and periosteum [14], [15], [16], [17], [18]. Interestingly, the area around forming bone is rich in mesenchymal stem cells. FGF-2 treatment of mesenchymal stem cells induces integrin α10 expression concomitant with induction of a chondrogenic phenotype [18]. Furthermore, in bone marrow-derived mesenchymal stem cells, α10 mRNA expression is observed as these cells form the chondrogenic niches needed to establish a bone marrow micro-environment [19]. In summary, several independent studies support a role for the integrin α10 subunit as a biomarker for chondrogenic stem cells [16].

α10β1 was identified in cartilage, but, as mentioned above, it is not exclusive to that tissue although its tissue distribution has not been exhaustively examined [14], [20]. Nor is it the only collagen-binding integrin expressed in chondrocytes, and for this reason we have used recombinant I domains from α10 and other integrins here, along with model cells (C2C12) that have been transfected with the relevant α subunit, pairing with the endogenously-expressed integrin β1 subunit. These various integrins have been applied to the Collagen Toolkits and short peptides derived from them, in either ELISA-like solid-phase binding assays (SPBA) or in label-free detection of C2C12 binding using the xCELLigence platform. This work has revealed constraints upon the ligand-binding properties that are unique to α10, examined further here by site-directed mutagenesis of the I domains and substitution of residues within the THPs.

## Results

### Functional analysis of α10 I domain by solid-phase binding assay

**Fig. 1:**
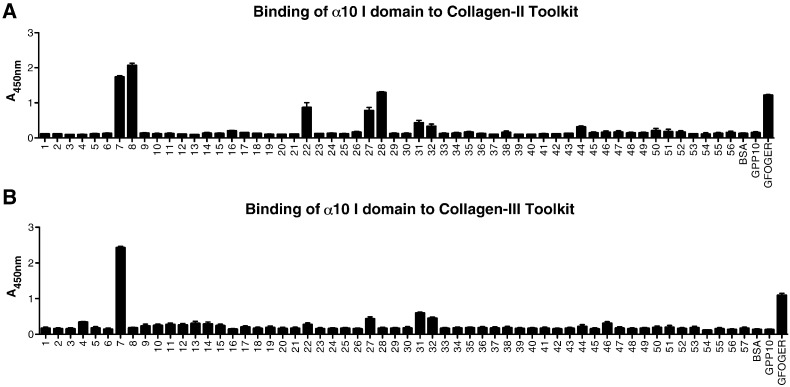
Binding of recombinant α10 I domain to Collagen Toolkits. 96-well plates were coated with peptide as described in Materials and methods, then α10 I domain (1 μg per well) was added and incubated for 1 h in the presence of either 2 mM MgCl_2_ or 2 mM EDTA (to chelate cations). BSA, GPP-10, and the known integrin ligand, GFOGER, were used as background, inert peptide scaffold, and positive control, respectively. Bound protein was detected as described in Materials and methods. **A**: binding to Toolkit II. **B**: binding to Toolkit III. Data obtained with EDTA were not different from those with BSA, and are not shown.

Ligand binding activity of the human recombinant GST-α10 I domain expressed in bacteria was first examined using conventional colorimetric 96-well plate SPBA. The α10 I domain was applied to the collagen II and III Toolkits in the presence or absence of Mg^2 +^, and was detected using anti-GST, as described in Experimental: data are shown in Fig. 1. The background response is indicated by wells coated with the control peptide, GPP10 (corresponding to the host sequence), or BSA. It should be noted that we have previously shown that a MIDAS-mutant α2 I domain GST fusion (T221A) showed no adhesion at all to Toolkit peptides [8], eliminating GST-mediated binding as a possible confounding factor. A THP containing GFOGER served as positive control in these and all other experiments. Data obtained in the presence of EDTA (all basal) are not shown for clarity. α10 I domain bound in Mg^2 +^-dependent manner, in rank order, to Toolkit peptides II-8, II-7, II-28, II-22, II-27, II-31, II-44 and II-32 (Fig. 1A) and to III-7, III-31, III-32, III-27, III-4 and III-46 (Fig. 1B), where binding to III-7 was by far the most prominent. Most of these reactive peptides contain a previously-identified integrin-binding motif, as GFOGER (II-28), GLOGER (II-7 and II-8), GMOGER (II-31, III-31 and III-32), and GVOGEA (II-27). Notably, GLOGEN occurs in III-7, a peptide that supports better adhesion of α10 I-domain than GFOGER, whilst III-4, which contains the relatively good α2β1-binding motif, GROGER [8], [10], supported slight or negligible binding.

### Binding of C2C12-α10β1 cells to Toolkit-II and III

**Fig. 2:**
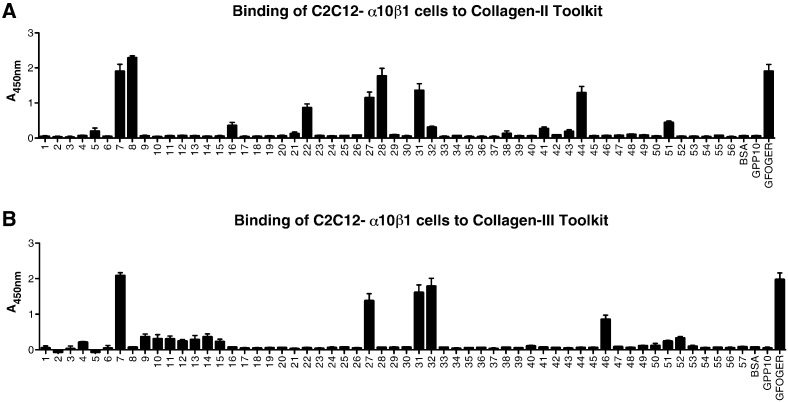
Binding of α10β1-transfected C2C12 cells to Collagen Toolkits. 96-well plates were coated with peptides as for Fig. 1, then C2C12-α10β1 cells (5 × 10^4^ per well) were added and incubated for 1 h in the presence of either 2 mM MgCl_2_ or 2 mM EDTA as above. **A**: binding to Toolkit II. **B**: binding to Toolkit III.

The mouse myoblast cell line, C2C12-α10β1 clone, expresses α10β1 as its sole collagen-binding integrin [16]. To investigate the binding of α10β1 to collagen in a cellular and whole integrin context, therefore, the adhesion of C2C12-α10β1 cells to Toolkits II and III was performed using two different approaches. First, SPBA was used, with lactate dehydrogenase to report the presence of adherent cells, after lysis, as described in Experimental. C2C12-α10β1 cells bound strongly, in a Mg^2 +^-dependent manner, in rank order, to peptides II-8, II-7, II-28, II-31, II-44, II-27, II-22, II-51, II-16 and II-32 (Fig. 2A). For Toolkit III, C2C12-α10β1 cells bound strongly to III-7, III-32, III-31, III-27 and less well to peptide III-46 (Fig. 2B). C2C12-α10B1 cells generally bound more prominently to those weaker peptides that supported just-observable binding of free I-domain. This may reflect avidity of the receptor-populated cell surface for the peptide-coated wells, or perhaps up-regulation of integrin affinity following receptor engagement. It is also possible that some cell binding reflects specific indirect interactions. For example, fibronectin, available either by intrinsic expression or from the serum supplement, binds well to II-44, and can also bind integrin αVβ3 [21].

**Fig. 3:**
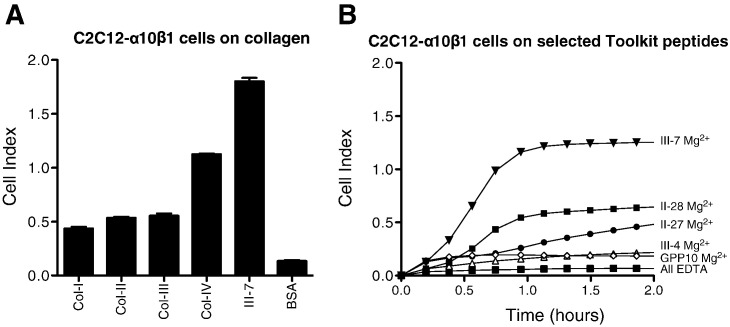
Binding of α10β1-transfected C2C12 cells to collagens and selected Toolkit peptides. Peptides or collagens were coated on ePlates as described, then cells were added in the presence of 2 mM Mg^2 +^ or EDTA, and Cell Index value was collected over 1 h. **A**: endpoint data are shown for collagens I, II, III and IV, with Toolkit peptide III-7 as positive control and BSA as background. Values collected with EDTA were identical to background, and are not shown. **B**: time courses are shown of adhesion to selected Toolkit peptides or GPP-10. In the presence of EDTA, all data obtained were close to background, and a single trace is shown.

Next, we used the impedance-based Acea xCELLigence system (Roche Applied Science) that allows label-free, real-time monitoring of cell adhesion to intact collagens and to selected positive Toolkit peptides. C2C12-α10β1 bound quite well to collagens I, II and III, better to collagen IV, with Peptide III-7 (containing GLOGEN) as a strongly positive control. For simplicity, endpoint data only of xCELLigence data are shown in Fig. 3A. Time courses are shown in Fig. 3B; III-7 supported the highest Cell Index, followed by II-28 (containing GFOGER) and II-27 (containing GVOGEA). Unlike other integrins tested to date, C2C12-α10β1 bound at the same level as the negative control peptide, to III-4 which contains GROGER, confirming the Toolkit data in Fig. 1, Fig. 2.

### Binding of alpha 10 I-domain and C2C12-α10β1 to short triple-helical motifs

**Fig. 4:**
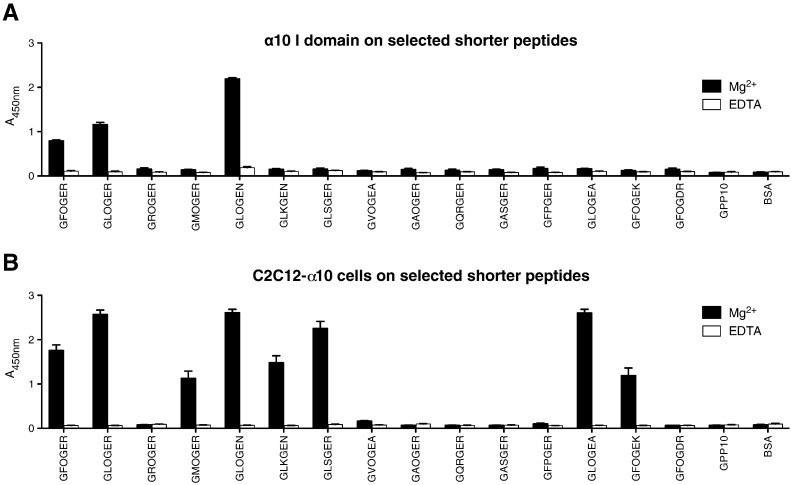
Binding of α10 I domain or C2C12-α10β1 cells to selected shorter peptides. 96-well plates were coated with the indicated peptides as for Fig. 1, Fig. 2, and binding of **A**: α10 I domain, or **B**: C2C12-α10β1 cells is shown as Mean ± SE. Data obtained with 2 mM Mg^2 +^ or EDTA are shown as black or white bars, respectively.

Using a set of short peptides, α10 I domain bound strongly to a THP containing GLOGEN and less well to GLOGER and GFOGER, shown in Fig. 4A. In contrast to the I domain, C2C12-α10β1 cells (Fig. 4B) bound strongly to GLOGEN, GLOGEA, GLOGER, and GLSGER, and less well to GFOGER, GLKGEN, GMOGER and GFOGEK. This series of experiments confirmed the absence of binding to GROGER. The collagens that contain these and related motifs are listed in Table 2. It is worth noting that the free α10β1 I domain binds as effectively as corresponding C2C12 cells to some motifs, notably III-7, but the lower affinity peptides are recognised better in the setting of the C2C12 cell. This effect is more apparent with the short triple-helical motifs rather than the longer Toolkit peptides.

### Comparison of C2C12-α10β1 binding of GRx′GEx″ with other integrins

**Fig. 5:**
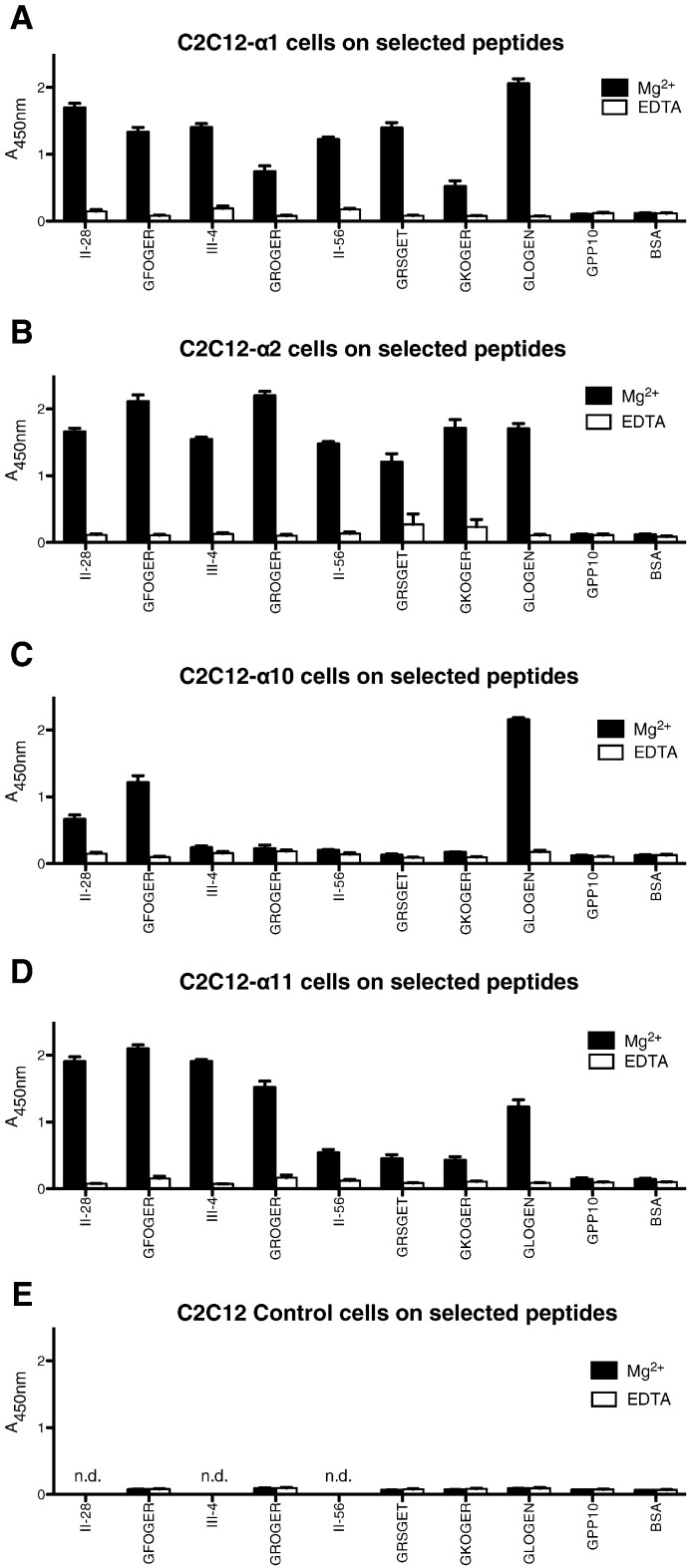
Binding of C2C12 cells expressing different collagen-binding integrins to selected peptides. 96-well plates were coated with the indicated peptides as for Fig. 1, Fig. 2, and binding of integrin-transfected C2C12 cells is shown as Mean ± SE. Data obtained with 2 mM Mg^2 +^ or EDTA are shown as black or white bars, respectively. **A**: C2C12-α1β1; **B**: C2C12-α2β1; **C**: C2C12-α10β1; **D**: C2C12-α11β1; **E**: parent, untransfected C2C12 cells.

In LDH-based SPBA (Fig. 5), transfected C2C12 cells, clones C2C12-α1β1, C2C12-α2β1, and C2C12-α11β1 showed strong and comparable binding to peptide III-4 (containing GROGER) where C2C12-α10β1 showed only background binding activity. Both C2C12-α1β1 and C2C12-α2β1 clones showed strong binding to peptide II-56 (GRSGET), whereas C2C12-α10β1 showed no binding. C2C12-α11β1 clones bound with intermediate affinity to II-56, suggesting that other local sequence in either receptor or collagen exerted a negative influence on α11β1 binding, but not sufficient to reduce activity to background levels. These data were confirmed by applying the same cells to short THPs containing GROGER and GRSGET, and to another motif, GKOGER, occurring only in collagen X and in the α2 chain of collagen I, which also contains a positively-charged x residue.

**Fig. 6:**
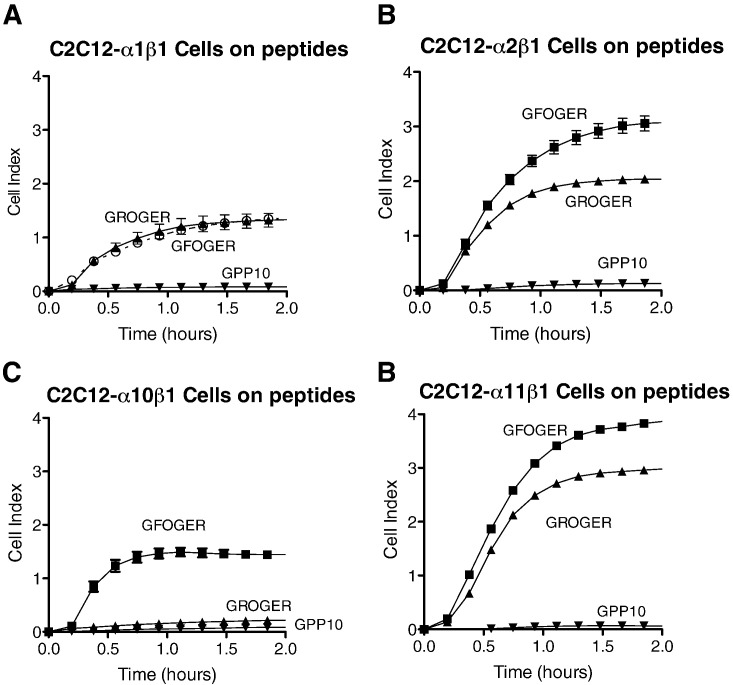
GFOGER- and GROGER-binding timecourse of C2C12 cells expressing different collagen-binding integrins. ePlates were coated with GFOGER, GROGER or GPP-10 as indicated, then the transfected cells were allowed to adhere for up to 1 h, as described for Fig. 3. As before, data obtained using EDTA were not different from background, and are not shown. **A**: C2C12-α1β1; **B**: C2C12-α2β1; **C**: C2C12-α10β1; **D**: C2C12-α11β1.

In real time xCELLigence assays testing the short peptide motifs, C2C12-α10β1 showed poor binding to the THP containing the GROGER sequence, of comparable level to the GPP10 negative control peptide (Fig. 6), whereas cells expressing the other three integrins bound quite well to GROGER compared to GFOGER.

### Gxx′GEx″ and the importance of residue x for α10β1 integrin

**Fig. 7:**
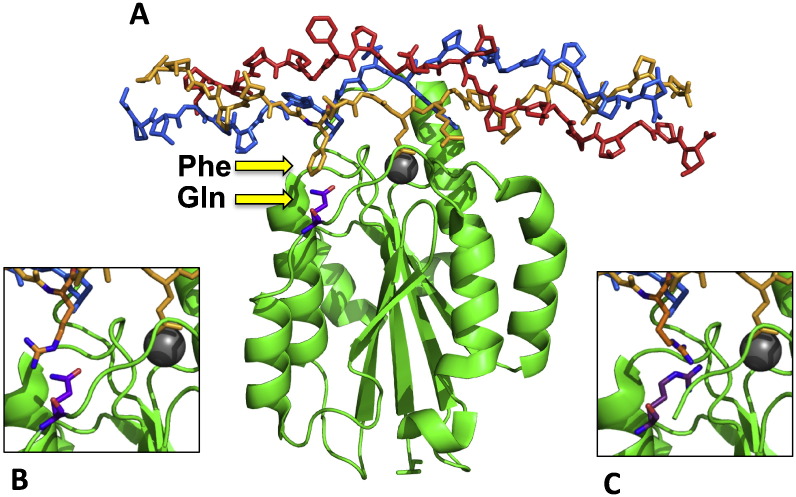
Modelling of the I domains to indicate the clash between R215 and GROGER. **A**: the main figure shows the crystal structure, 1DZI, a complex between GFOGER and the α2 I domain. The phenylalanine of the peptide is closely apposed to glutamine-215 in α2 I domain, indicated by arrows. **Inset B** shows the same structure with arginine modelled into the peptide to represent GROGER. **Inset C** shows the corresponding arginine substitution of both peptide and I domain, and the resulting charge–charge clash.

We re-examined the PDB structure, 1DZI, a co-crystal of the collagen hexapeptide motif, GFOGER, with the α2 I domain, to establish the contacts between the peptide x residue and the I domain surface. In that context, the peptide F residue makes some contact with Q215 of the α2 I domain (Fig. 7). Replacing F with R (i.e. generating GROGER) is tolerated by α1, α2, and α11 I domains, although no crystal structures exist for these integrin complexes. Alignment of the I domain sequences (Table 1, below) revealed an arginine residue at position 215 of the α10 I domain instead of the glutamine that is conserved in all other members of the family. We proposed that the R215 in the α10 I domain L2 loop would cause repulsion with the x positive charge (the first R) of GROGER.

**Fig. 8:**
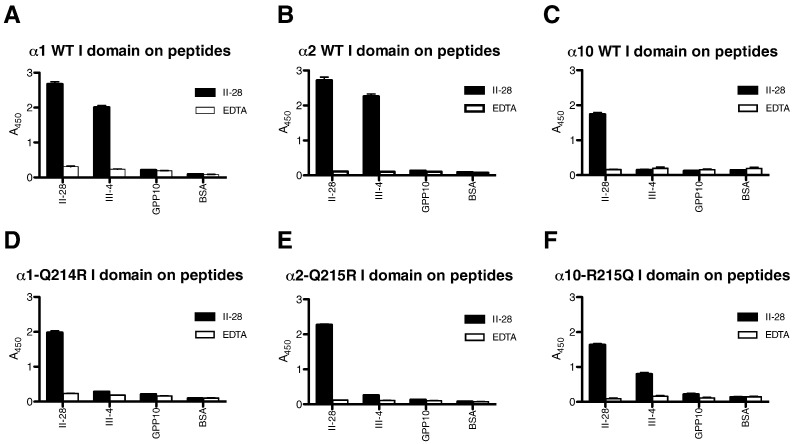
Arginine substitution of I domain surface reduces binding to Toolkit peptide III-4. Wild type I domain from α1, α2 and α10 were allowed to adhere to Toolkit peptide III-4 that contains GROGER, as described in Fig. 1, and binding is shown in **A**, **B** and **C**, respectively. In **D** and **E**, the natural glutamine residues of α1, Q214, and of α2, Q215, was replaced with arginine. In **F**, the natural arginine, R215, of α10 was replaced with glutamine. The data show that the engineered charge clash in α1 and α2 abolishes binding, but that the natural clash in α10 is rescued by R215Q substitution.

**Fig. 9:**
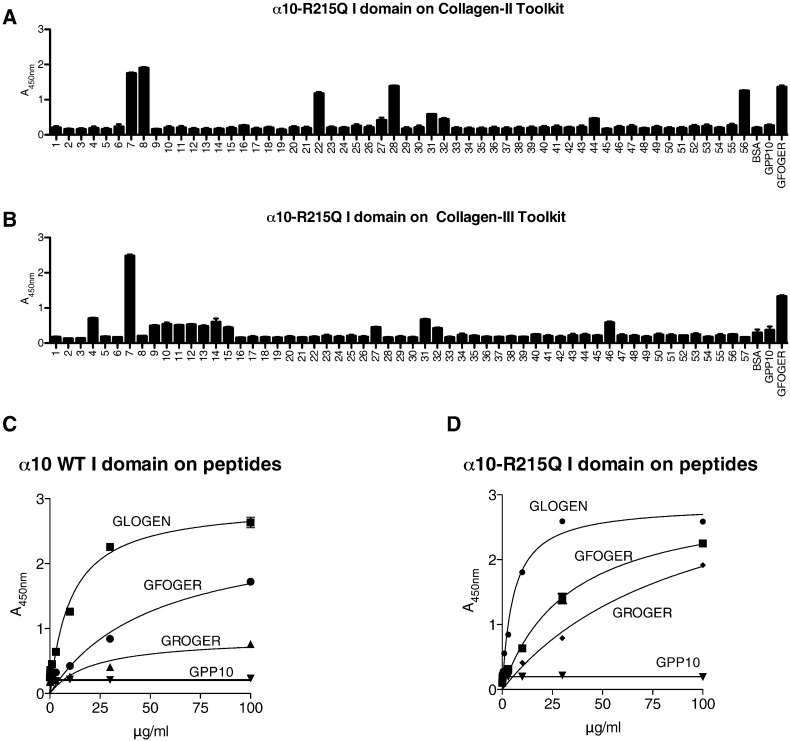
Binding of α10 R215Q to Toolkit and short peptides. 96-well plates were prepared as for Fig. 1. **A**: binding to Toolkit II, **B**: binding to Toolkit III. Note enhanced binding to II-56 and III-4, that contains GRSGET and GROGER, relative to data in Fig. 1. **C** and **D** show concentration-dependence of binding to GLOGEN, GFOGER and GROGER of wild type and of R215Q α10 I domain. The data show enhanced binding of R215Q to GROGER but not GLOGEN.

To test this hypothesis, we replaced the corresponding Q214 and Q215 in α1 and α2 I domains, respectively, with R, as in α10, and we replaced the R215 in the α10 I domain with Q, as in α1 and α2. The expressed mutants of α1-Q214R and α2-Q215R I domains failed to bind to GROGER, whereas the α10 I domain R215Q mutant showed better binding to GROGER (Fig. 8, and see Fig. 9C and D.)

### Functional analysis of α10-R215Q I domain by SPBA

Ligand binding activity of the human recombinant GST-α10-R215Q I-domain expressed in bacteria was examined using conventional colorimetric 96-well SPBA. The I domain was first applied to the collagen II and III Toolkits in the presence or absence of Mg^2 +^, detected using anti-GST as before, and data are shown in Fig. 9A and B. α10-R215Q I domain binding was Mg^2 +^-dependent, reproducing the wild type Toolkit II data shown in Fig. 1A. For Toolkit III, α10-R215Q I domain bound well to III-7, as for wild type I domain, with III-4 binding more prominently than the wild type (Fig. 1B). Notably, though, the mutant α10 I domain bound very well to peptide II-56, that contains the sequence GRSGET, also found to be a good ligand for α1β1 (Fig. 5).

### Binding kinetics of α10 and α10-R215Q I domains to short triple-helical peptides

GFOGER, GLOGEN and GROGER have previously been described as good binding motifs for integrin α2β1. We used the three peptides here to evaluate the binding capacity of wild type α10 I domain and its engineered R215Q mutant. The data showed saturation binding of both constructs to GLOGEN, with K_D_ on the order of 10 μg/ml (Fig. 9C and D). GFOGER was a good ligand for both recombinant proteins, but did not saturate at up to 100 μg/ml, showing a lower K_D_ of about 50 and 30 μg/ml for WT and R215Q respectively. The wild type α10 I domain bound poorly to GROGER, with K_D_ not estimable, but ≫ 100 μg/ml. In marked contrast, R215Q bound quite well to GROGER, with K_D_ of about 50 μg/ml, the same order as wild type for GFOGER.

## Discussion

In the present work, we establish the binding selectivity of α10β1 for collagenous ligands using Toolkit and short THPs, as used here and by others previously. Our work indicates that α10β1, like α1β1, can use GLOGEN (found only in collagens III and XXII) as a selective ligand in preference to GFOGER. No other peptide (or shorter derivatives) from Toolkit III approached the binding activity of III-7/GLOGEN. In the setting of cartilage, collagen III, although enriched in the pericellular environment, is much less abundant than collagen II [22], and its expression increases with age or with the onset of osteoarthritis [23]. However, collagen II, the main fibrillar collagen of cartilage, lacks GLOGEN but contains GLOGER which binds α10β1 better than GFOGER, second only to GLOGEN, and GVOGEA which is shown here to be a moderately good ligand for α10β1. In these respects, α10β1 displays a similar collagen ligand selectivity to α1β1. α10β1 is reported to be a better ligand for the non-fibrillar collagens, IV and VI, than for collagens I, II and III, although it binds the prominent cartilage collagens, II, XI and the FACIT, collagen IX. Collagen IV is rich in GLOGEx″ motifs, and also contains the degenerate α1β1-binding site, described by Kuhn and colleagues, assembled from a specific arrangement of D and R residues in its α1 and α2 chains [24], [25]. The cartilage collagens similarly lack GLOGEN but do contain other GLOGEx″ motifs, along with both GFOGER and GVOGEK, suggesting that these might also contribute to the binding and regulation of chondrocytes by α10β1. The short chain collagen X, prominent in hypertrophic cartilage [26], is unusual. Although it contains four possible integrin-binding motifs, two, GROGER and the hitherto-unreported GKOGER, are compromised in the context of α10β1 by their positively-charged residue at position x, as we show here. Two potential sites remain, GFOGEK and GFOGEM, and the former is now shown to bind α10β1. Collagen VI is under-researched because of the difficulty of assembling its most widely-expressed form, an α1α2α3 heterotrimer. However, it contains several possible motifs, including GFOGEK and GLOGEK, both of which might be good α10 ligands. It is interesting that both the weak ligand GAOGER and GFOGEK align with GYx′GEx″-containing motifs in the α1(VI) chain; the possibility that Y might substitute for the hydrophobic F or L residues has not been tested, and may improve the affinity of such sites.

The only other peptide from Toolkit II that shows good affinity for α10β1 and α10 I domain is II-22, that contains no obvious integrin motif, but is a good ligand for the discoidin domain receptors, DDR1 and DDR2, and for the ECM components SPARC and von Willebrand factor. It may well be that some of these proteins augment the regulation of chondrocytes, by co-localising with α10β1, although this is beyond the scope of the present study. A potential role for other collagen receptors was revealed by the use of a blocking α10 antibody [16], which did not prevent cell adhesion. The major DDR-binding site in the fibrillar collagens, the sequence GVMGFO [27], [28], is axially separated from the nearest integrin-binding motif, GLOGER in collagens I and II, by 12 nm or so, on the order of one receptor diameter, lending credence to this concept.

By sequence alignment and molecular modelling, we establish that the α10 I domain differs from other members of the family by the presence of a positively-charged residue, R215, at the site where the long hydrophobic residue x (of Gxx′GEx″) would interact with the I domain surface. Other I domains that lack such a positive residue at this site bind GROGER well, and presumably the long aliphatic stem of the collagen arginine sidechain can support a hydrophobic interaction with the I domain surface. This is not the case with native α10β1 where the electropositive R215 will repel GROGER. This α10 residue is conserved in many other species; rat or mouse [29], dog [30] and sheep [31], animals that are frequently used in models of experimental arthritis; cow, a large-scale source of cartilage [32]; and zebrafish [33], [34], [35], a widely-used developmental model. All contain this critical arginine residue. We show here using site-directed mutagenesis that neutralising the surface charge of α10 I domain by replacing R215 with a glutamine residue (of similar length, but neutral) substantially improves its ability to bind GROGER-containing Toolkit peptide III-4, and we show in a complementary experiment that introducing an arginine residue into the equivalent site in α1 or α2 I domains causes the collapse of binding to GROGER, inducing α10-like binding specificity. A similar effect is especially prominent with Toolkit peptide II-56 which contains the intermediate-affinity motif, GRSGET, a sequence unique to collagen II that is negative for wild type α10 I domain, but strongly binds R215Q.

Why would collagen II differ in this respect? Nine conserved integrin-binding sites are distributed across the fibrillar collagens (see Table 4); Site 1 carries a unique, conserved, high-affinity motif [8], [10] GROGER, present in mammalian collagens I and III. Site 2, GLOGEN, is unique to collagen III, and is a high-affinity site for integrins α1β1and α10β1. Sites 3, 5 and 6 also contain high- or good-affinity motifs, and the remainder are conserved but of lower affinity. Site 1 cannot engage α10β1, the major chondrocyte integrin [4], in any fibrillar collagen, nor can any integrin bind Site 1 in collagen II, where the motif is defective. Since the integrin complement of vertebrates is competent to bind several other sites in collagen II, and can presumably be regulated by engagement with these sites, the peculiar relationship between Site 1 and α10β1 must reflect a function of collagen, rather than of the integrin.

The collagen fibre adopts its canonical quarter-stagger assembly, with 300 nm triple-helical tropocollagen monomers laid end-to-end with an axial gap of ~ 35 nm between. The 67 nm axial offset between adjacent monomers defines the D-period observed in transmission electron micrographs of native collagen fibres. This simple model allows side-by-side location of binding sites in adjacent collagen molecules to be considered, and reveals that Site 1 in collagen II (GROGER) and Site 9 (GRSGET), both unable to bind α10β1, are almost perfectly co-located in the D-period overlap region. Thus, this specific axial locus in the collagen fibre cannot be ligated by the principal chondrocyte integrin, suggesting that occlusion of this specific site by α10β1 ligation is not tolerated and there is a need to preserve access to this region of the collagen II fibre for other activities. Similarly, this locus in collagens I and III is protected against α10β1 binding, implying that in α10β1-expressing tissues, i.e. in cartilage, it is also preserved from integrin ligation.

Since a collagen II orthologue is considered to precede the other fibrillar collagens in evolutionary history [36], GROGER may have arisen in the primordial α1(II) chain, but evolutionary pressure has preserved the GROGER motif in collagens I and III, but not II, which now contains the silent GKAGER. The ghost shark (*Callorhyncus milii*) is a cartilaginous fish whose evolution diverged prior to the emergence of the bony fish and other vertebrates [36]. Site 1 in its fibrillar collagens II and III contains GKSGEO and GROGEO, respectively, both either α10β1-repellent or not competent to bind any integrin (though untested), whilst shark collagen I retains the primordial GROGER. GKOGER and GRSGET are absent. Shark collagen II lacks any GLOGEx″ motif that might bind α10β1, but contains GIOGER, also untested, in conserved Sites 3 and 5 that may well be α10β1-positive. Shark collagen III contains two copies of GLOGER and one of GLSGER (at a non-conserved site between Sites 4 and 5), both motifs shown here to be α10β1-positive. These binding sites are summarised in Table 4.

However, further inspection of the cartilaginous fish genome sheds little direct light: the ghost shark contains orthologues of α1β1, α2β1 and α11β1, but appears to lack α10 [37], confirmed here using each human integrin sequence to search the *C. milii* genome. These shark integrins all contain the critical, permissive, Glu residue, so that they are predicted to bind Site 1 in collagens I and III. In contrast, bony fish (exemplified by zebrafish, *Danio rerio*) resemble most tetrapods in terms of the distribution and identity of collagen-binding integrins and integrin-binding sites in the collagens.

This suggests that co-evolution of collagens I and III with the calcified skeleton may have occurred, and required the prohibition of chondrocyte binding to Site 1 in collagens I and III, along with the absence of any cellular binding to Site 1 in collagen II. One might speculate that these properties would maintain access of osteoclasts and their precursors to the nearby OSCAR motifs in collagen II, GAOGPQGFQ in D1 and the collagen II-specific GAOGASGDR motif [38], [39] in D5, to promote its resorption, or might allow access of regulatory species such as decorin or fibromodulin [40] to their binding sites near the KGHR fibre-crosslinking locus in D1 and D4. Access of bone-forming cells to both sites in the nascent collagens I and III might be crucial as maturing cartilage is resorbed and replaced by bone during endochondral ossification and skeletal development. Consistent with these ideas, the Lundgren-Åkerland group [16], [41] has reported disorganisation of the cartilage growth plate and of the collagen fibre network in α10-null mice. The unique sequence of collagen II and the unique specificity of α10β1 might therefore represent an evolutionary strategy rather than simple coincidence.

The increased expression of collagen III has been proposed as a repair response after damage or disease in cartilage [23]. The inability of the major chondrocyte integrin, α10β1, to engage with the major integrin locus in collagen III, GROGER, suggests instead that this collagen should rather be regarded as a marker for cartilage dysfunction or resorption.

The three α10-repellent motifs identified here, GROGER, GKOGER and GRSGET, summarised in Table 3, occur in loci conserved across species with their human collagen counterparts, and so we anticipate that animal models where α10β1 may be important, for example in modelling diseases of cartilage and skeletal development, will faithfully reflect the spectrum of integrin-binding activity.

## Materials and methods

### Reagents

Unless stated otherwise, reagents were from Sigma (Poole, Dorset, UK).

#### Peptides

Toolkit peptides, as C-terminal amides, were synthesised on TentaGel R-Ram resin using either an Applied Biosystems Pioneer peptide synthesiser as described previously [8]. Shorter peptides were made using the same Fmoc chemistry in a CEM Liberty microwave-assisted peptide synthesiser. In either case, fractions containing homogeneous product were identified by analytical HPLC on an ACEphenyl300 (5 mm) column, characterised by MALDI-TOF mass spectrometry, pooled and freeze-dried. In both peptide formats, the variable primary collagen motif (guest sequence) was flanked by GPC [GPP]- and - [GPP]_5_GPC host peptides, which confers triple-helical form on the shorter peptides.

#### Antibodies

Mouse anti-glutathione S-transferase antibody (Horseradish peroxidase-conjugated) was from GE Healthcare (cat. no. RPN1236).

#### Plasmids

Recombinant human α10 I domain-encoding plasmid (pGEX-2T-α10 I domain) was cloned using standard procedures [12].

#### Site-directed mutagenesis

pGEX-2T-α1, α2 and α10 I were used to generate the Q to R or R to Q mutations using QuikChange PCR kit from Stratagene (200518).

### Methods

#### I domain expression and purification

To express α2 wild type and mutant I domains, a 100-ml overnight culture of transformants (Origami strain) was used to inoculate 1 L of Luria broth containing 100 μg/ml ampicillin, 15 μg/ml Kanamycin and 12.5 μg/ml Tetracyclin. The culture was grown for 2 h at 37 °C and then induced at room temperature for 4 h with isopropyl β-d-thiogalactopyranoside (0.1 mM, Melford Laboratories, UK, #MB1008). Cells were harvested by centrifugation at 4500*g* for 20 min, and pellets for GST-fusion were resuspended in 10 ml Dulbecco's phosphate-buffered saline, containing 1 tablet of protease inhibitor cocktail (Roche) and 5 mg of lysozyme (Fluka). Suspensions were sonicated and Triton X-100 was added to 1% (v/v). Suspensions were mixed on a roller for 15 min at room temperature, then centrifuged at 18,000 ×* g* for 20 min, and supernatants were pooled. The lysate was passed down a glutathione-agarose column equilibrated in Tris-buffered saline (20 mM Tris–HCl and 150 mM NaCl, pH 7.5); the column was washed with 10 volumes of Tris-buffered saline containing 1 M NaCl and 1% (v/v) Triton X-100, and the glutathione S-transferase–I domain fusion proteins were eluted with 10 mM glutathione reduced in 50 mM Tris–HCl (pH 8.0). The proteins were then dialyzed against Tris-buffered saline and concentrated using a Microcon-3 (Amicon, Stonehouse, Gloucestershire, UK). I domain purity and degradation were checked using 10% SDS-PAGE and Western blotting. Nitrocellulose blots were probed with horseradish peroxidase-conjugated anti-glutathione S-transferase polyclonal antibody (GE Healthcare).

#### I domain binding assay

I domain adhesion was determined colorimetrically, as described [42], here termed solid phase binding assay (SPBA). Peptides were coated at 10 μg/ml for 1 h at 22 °C on Immulon-2 HB 96-well plates (Thermo Life Sciences, Basingstoke, UK), and blocked for 1 h with 200 μl of TBS containing 50 mg/ml bovine serum albumin. Wells were washed four times with 200 μl of the adhesion buffer (TBS with 1 mg/ml bovine serum albumin) before adding 100 μl of adhesion buffer containing 10 μg/ml of recombinant GST I domains in the presence of either 2 mM MgCl_2_ or EDTA for 1 h at room temperature. Wells were washed five times with 200 μl of adhesion buffer containing MgCl_2_ or EDTA, before adding 100 μl of adhesion buffer containing the anti-GST-HRP conjugate at 1:10,000 dilution for 1 h at room temperature. After washing, colour was developed using an ImmunoPure TMB Substrate Kit (Pierce) according to the manufacturer's instructions.

#### Cell culture and adhesion

The mouse myoblast cell line, C2C12, (control and transfectants, C2C12-α1β1, C2C12-α2β1, C2C12-α10β1 or C2C12-α11β1) was maintained in RPMI medium containing 10% fetal bovine serum, 2 mM glutamine, 100 IU/ml penicillin, 100 μg/ml streptomycin, and 2.5 μg/ml amphotericin. Cells were harvested by centrifugation, washed, and suspended at 0.5 × 10^6^/ml in Tris-buffered saline supplemented with 5.5 mM glucose and either 2 mM EDTA or 2 mM MgCl_2_. SPBA was performed as follows: 100 μl of the cell suspension was added to peptide-coated wells [42] at 20 °C for 60 min. Cell adhesion was determined colorimetrically after lysis, using a lactate dehydrogenase (LDH)-based cell detection kit (Roche Diagnostics) according to the supplier's instructions.

#### Real time binding using xCELLigence technology

To quantitate C2C12-α10β1 binding to collagen peptides, we used the impedance-based xCELLigence system (Acea) that allows label-free, dynamic monitoring of cell adhesion in real-time [12]. The assay system expresses the adhesion-dependent rise in well impedance in units of cell index (CI), defined as (R_n_ − R_b_) / 15; where R_n_ is the electrical impedance of each cell-containing well and R_b_ is the background impedance of the well with medium alone

Briefly, C2C12 cells were seeded at 2.5 × 10^4^ cells/well in an E-plate (Roche Applied Science) pre-coated with peptides as for SPBA, and CI was measured for up to 2 h.

#### Presentation of data

Values shown in all figures are mean ± s.d.

## Funding

This work was supported by British Heart Foundation programme grants, RG/15/4/31268 and RG/09/003/27122, and by a Wellcome Trust Biomedical Resource grant, 094470/Z/10/Z.

## Figures and Tables

**Table 1:** Restrictive Arginine residue in α10 I domain contrasts with Glutamine in other I domains.

α2	VVFNLNTYKT KEEMIVATSQ TS**Q**YGGDLTN TFGAIQYARK YAYSAASGGR

α11	HEFHLNDYRS VKDVVEAASH IE**Q**RGGTETR TAFGIEFARS EAFQK--GGR
α10	HEWSLGDFRT KEEVVRAAKN LS**R**REGRETK TAQAIMVACT EGFSQSHGGR
α1	HEFNLNKYSS TEEVLVAAKK IV**Q**RGGRQTM TALGIDTARK EAFTEARGAR

*	

Human integrin I domain partial sequence, showing the alignment of critical Q/R residue (indicated * on lower line).

**Table 2:** Distribution of relevant integrin motifs in human collagens.

Motif	Collagen type	Motif	Collagen type
GFOGER	I, II, IV, XI	GAOGER	II, III, IV, VI, VII, IX
GLOGER	I, II, VII	GQRGER	I, II, V
**GROGER**	I, III, VII	GASGER	I, XXIII
GMOGER	I, II, III, V, IX	GFPGER	Model
GLOGEN	III, XXII	GLOGEA	IV, XI, XXVII
GLKGEN	III	GFOGEK	IV, VI, X,
GLSGER	III	GFOGDR	V
GVOGEA	II	GFOGEK	IV, VI, X
**GRSGET**	II	**GKOGER**	I (α2 chain), X

The collagens containing motifs of interest are identified above. GFPGER is a model peptide, not found in collagens, although a related motif, GLPGER, occurs in a bacterial collagen [43] where prolyl hydroxylase is absent. GFOGDR, present in collagen V, but not in II and III from which the Toolkits were derived, was included in the study, since it contains an aspartate residue that might ligate integrin MIDAS, as in the α1β1 site in collagen IV [25]. α10β1-repellent motifs are shown in **bold text**.

**Table 3:** Alpha10-negative integrin motifs in collagens of different species.

	GROGER	GKOGER	GRSGET
Human	α1(I), α2(I); α1(III); α1(VII); α1(X)	α2(I); α1(X)	α1(II)
Rat	α1(I), α2(I); α1(III); α1(VII); α1(X)	α2(I)	α1(II)
Mouse	α1(I), α2(I); α1(III); α1(VII); α1(X)	α2(I)	α1(II)
Dog	α1(I), α2(I); α1(III); α1(VII); α1(X)	–	α1(II)
Sheep	α1(I), α2(I); α1(III); α1(VII); *α1(X): CROGER*⁎	–	α1(II)
Cow	α1(I), α2(I); α1(VII); *α1(III): GROGPR;* *α1(X): CROGER*⁎	α2(I)	α2(I); α1(II)
Zebrafish	α1a(I); α1b(I); *α2(I): GROGKOGDR* *α1(VII): GROGEK*	α1a(I); α1b(I); α1(VII)	*α1(II): GRSGES*
Chick	α2(I); *α1(I): GROGQR;* *α1(III): GRNGDR*	α1(I); α2(I)	*α1(II): GRSGEO*

The table shows the occurrence of integrin-binding motifs across different collagens and species that are predicted not to bind integrin α10β1 by virtue of a positively-charged x residue in the canonical GxoGExʺ motif. Italic text indicates loci that are not fully conserved with human sequence.

⁎ The Uniprot sequence from sheep and bovine collagen Colα1(X), CROGER, breaks the Gxx′ triplet rule, and may need re-evaluation.

**Table 4:** Integrin sites in human, shark and zebrafish collagens I, II and III.

Locus (Helix no)		**1** (61)	**2** (112)	**3** (127)	4 (328)	**5** (502)	**6** (550)	7 (787)	8 (811)	9 (991)
Toolkit# II/III		4	7	7/8	18/19	28	31/32	44	46	56
Human	α1(I) α2(I) α1(II) α1(III)	GROGER GROGER – GROGER	– – – GLOGEN	GLOGER GLOGER GLOGER GAOGER	– – GAOGER –	GFOGER – GFOGER GAOGER	GMOGER GLOGER GMOGER GMOGER	GQRGER – GQRGER –	GASGER – GASGDR GLSGER	– – GRSGET *GNRGER*
Shark	α1(I) α2(I) α1(II) α1(III)	GROGER – *GKSGEO* *GROGEO*	– – – *GLKGEV*	GLSGER GLSGER GIOGER GLOGER	GAOGER – – GLOGER	GLSGER – GIOGER –	GMOGER GIOGER GMOGER GMOGER	– – GQRGER –	– – – GROGER	*GRTGEV* – *GNFGES* *GTRGES*
Zebra-fish	α1(I) α2(I) α3(I) α1(II)	GROGER – GROGER –	*GAOGEN* – – –	GLOGER GLOGER GLOGER GLOGER	GAOGER – GAOGER –	GFOGER – GFOGER GFOGER	GMOGER GMOGER GMOGER GMOGER	GQRGER – GQRGER GQRGER	– – – –	*GRSGED* – *GRSGED* *GRSGED*

Nine conserved integrin-binding sites are found in the Clade A fibrillar collagens, I, II and III [36]. Zebrafish lacks collagen III, its function in skin being assumed by an ABC heterotrimer of the three collagen I α-chains.

Bold numerals indicate the higher-affinity sites. Helix numbering relates to the start of the conventional, 1014-residue collagen helix, as occurs in human collagens I and II. The value given is the position of the first Gly in the integrin-binding motif. For further orientation, the two cross-linking motifs, KGHR, start at 87 and 930, and the mammalian MMP-cleavage site (Gly ~ Leu or Gly ~ Ile) at 775.

A dash (–) represents sequence that is only partially conserved, and assumed not to bind integrins; italic text indicates closer conservation, but these motifs are untested.
